# C-type natriuretic peptide analog treatment of craniosynostosis in a Crouzon syndrome mouse model

**DOI:** 10.1371/journal.pone.0201492

**Published:** 2018-07-26

**Authors:** Greg Holmes, Lening Zhang, Joshua Rivera, Ryan Murphy, Claudia Assouline, Lorraine Sullivan, Todd Oppeneer, Ethylin Wang Jabs

**Affiliations:** 1 Department of Genetics and Genomic Sciences, Icahn School of Medicine at Mount Sinai, New York, New York, United States of America; 2 BioMarin Pharmaceutical, Novato, California, United States of America; University of California Davis, UNITED STATES

## Abstract

Activating mutations of fibroblast growth factor receptors (FGFRs) are a major cause of skeletal dysplasias, and thus they are potential targets for pharmaceutical intervention. BMN 111, a C-type natriuretic peptide analog, inhibits FGFR signaling at the level of the RAF1 kinase through natriuretic peptide receptor 2 (NPR2) and has been shown to lengthen the long bones and improve skull morphology in the *Fgfr3*^*Y367C/+*^ thanatophoric dysplasia mouse model. Here we report the effects of BMN 111 in treating craniosynostosis and aberrant skull morphology in the *Fgfr2c*^*C342Y/+*^ Crouzon syndrome mouse model. We first demonstrated that NPR2 is expressed in the murine coronal suture and spheno-occipital synchondrosis in the newborn period. We then gave *Fgfr2c*^*C342Y/+*^ and *Fgfr2c*^*+/+*^ (WT) mice once-daily injections of either vehicle or reported therapeutic levels of BMN 111 between post-natal days 3 and 31. Changes in skeletal morphology, including suture patency, skull dimensions, and long bone length, were assessed by micro-computed tomography. Although BMN 111 treatment significantly increased long bone growth in both WT and mutant mice, skull dimensions and suture patency generally were not significantly affected. A small but significant increase in the relative length of the anterior cranial base was observed. Our results indicate that the differential effects of BMN 111 in treating various skeletal dysplasias may depend on the process of bone formation targeted (endochondral or intramembranous), the specific FGFR mutated, and/or the specific signaling pathway changes due to a given mutation.

## Introduction

Skeletal dysplasias comprise a diverse group of disorders. The skeleton forms via both intramembranous ossification, by which osteoblasts differentiate directly from mesenchyme to form bone, and endochondral ossification, by which a cartilage template of individual bones is first established and then replaced by osteoblasts to form the final bone. The flat bones of the skull form via intramembranous ossification, while the base of the skull, vertebrae, and long bones of the limbs form via endochondral ossification. Common skeletal dysplasias include craniosynostosis, in which the sutures separating the skull bones fuse prematurely [1], and chondrodysplasias resulting in dwarfism [2]. Activating mutations of fibroblast growth factor receptors (FGFRs) are a major cause of skeletal dysplasias, including syndromic craniosynostosis and chondrodysplasias [3]. For many of these conditions complex surgical intervention is the only therapeutic strategy, and thus pharmacological attenuation of FGFR activity is an attractive potential alternative for treatment of these skeletal dysplasias.

C-type natriuretic peptide (CNP) binds the guanyl cyclase natriuretic peptide receptor 2 (NPR2) and activation of NPR2 results in the inhibition of the FGFR signaling pathway at the level of the RAF1 kinase [4, 5]. Total or chondrocyte-specific genetic deletion of *Nppc* (the gene encoding CNP) [6–8] or *Npr2* [8, 9] in mice severely impairs endochondral ossification, resulting in dwarfism characterized by shortening of the vertebrae, long bones, and skull. Conversely, overexpression of CNP in chondrocytes using a collagen 2 promoter sequence [10] or systemically from the liver using a human serum amyloid P promoter [11] results in skeletal overgrowth. Chondrocyte-specific or systemic overexpression of CNP from these transgenic alleles was also able to ameliorate the dwarfism phenotype of the transgenic *Fgfr3*^*ACH/+*^ achondroplasia mouse model [10, 12], in which *Fgfr3*^*G380R*^ expression was also targeted to cartilage using a collagen 2 promoter sequence [13]. Loss-of-function mutations in the human *NPR2* gene result in short stature and the dwarfism syndrome acromesomelic dysplasia, Maroteaux type [14, 15]. CNP or modified CNP analogs are considered a potential therapeutic strategy for the treatment of human chondrodysplasias [16, 17].

BMN 111 is an analog of CNP currently in clinical development for achondroplasia. BMN 111 exhibits the pharmacological activity of CNP but has an extended half-life, allowing for once-daily subcutaneous delivery. It has been shown to reduce the adverse growth plate cartilage phenotype and lengthen the long bones in both the *Fgfr3*^*Y367C/+*^ thanatophoric dysplasia mouse model [18] and the *Fgfr3*^*ACH/+*^ achondroplasia mouse model [19]. Skull dysmorphology was also reduced in the *Fgfr3*^*Y367C/+*^ mice [18].

Crouzon syndrome is a debilitating autosomal dominant syndromic condition characterized by craniosynostosis, maxillary hypoplasia, and exophthalmos. The coronal sutures of the neurocranium are most commonly synostosed, but other sutures can also be affected, and the head shape is typically short and broad, with a shortened skull base. Other consequences include obstructive sleep apnea, hydrocephalus, and adverse psychosocial effects [20, 21]. This syndrome is caused by a variety of activating mutations of *FGFR2* that render the receptor constitutively active [22, 23]. In a mouse model of Crouzon syndrome with the *Fgfr2c*^*C342Y/+*^ mutation, extensive coronal suture fusion occurs by two weeks of age and is complete by four weeks [24, 25]. Pharmacological inhibition of FGFR tyrosine kinase activity can reduce the coronal suture fusion in calvarial organ culture derived from this model [25]. In this study we evaluated the efficacy of BMN 111 for the correction of craniosynostosis and the more general craniofacial dysplasia in this mouse model during postnatal development.

## Materials and methods

### Mice

Crouzon syndrome *Fgfr2c*^*C342Y/+*^ model mice [24] were maintained on the outbred CD1 background. Experimental litters were generated by breeding *Fgfr2c*^*C342Y/+*^ males with CD1 females (Charles River Laboratories, Wilmington, MA). Pups were genotyped by PCR of tail DNA to identify heterozygous *Fgfr2c*^*C342Y/+*^ mutants and litter-matched, wild type (WT; *Fgfr2*^*+/+*^) controls. Water and food (5053 Picolab Rodent Diet 20, LabDiet, St. Louis, MO) were available *ad libitum*, and mice were maintained on a 12:12 hour light:dark cycle. All use of mice was in compliance with animal welfare guidelines approved by the Icahn School of Medicine at Mount Sinai Institutional Animal Care and Use Committee.

### Immunohistochemistry

For detection of NPR2 and RUNX2 in untreated animals, heads and hindlimbs of newborn mice were fixed in 4% paraformaldehyde (PFA) overnight, washed in phosphate-buffered saline (PBS), and placed in 30% sucrose/PBS overnight. They were then embedded in Tissue-Tek O.C.T. Compound (Sakura, Japan) in square plastic molds (Shandon Peel-A-Way Disposable Embedding Molds, S-22, ThermoFisher Scientific), sectioned on an Avantik QS11 cryostat at a thickness of 10 μm, and stored at -80°C. For protein detection, sections were dried, permeabilized in 0.3% Triton X-100/Tris-buffered saline (TBS) for 15 minutes, and treated with 0.6% hydrogen peroxide in methanol for 20 minutes. Sections were blocked in 10% goat serum/TBS and treated with primary antibodies overnight at 4°C. After washing in TBS, biotinylated anti-rabbit secondary antibodies (BA-100, 1/200, Vector Laboratories, Burlingame, CA) were applied. Bound antibodies were visualized using the ABC HRP (horseradish peroxidase) Kit (PK-4001, Vector Laboratories, Burlingame, CA) and the DAB Peroxidase Substrate Kit (SK-4100, Vector Laboratories, Burlingame, CA), as described by the manufacturer. Primary antibodies used were rabbit anti-NPR2 (ab14357, 1/200, Abcam, Cambridge, MA) and rabbit anti-RUNX2 (HPA022040, 1/200, Sigma, St. Louis, MO). Normal rabbit IgG serum (I-1000, 1/10,000, Vector Laboratories, Burlingame, CA) was used for negative control staining.

### Experimental design for BMN 111 treatment

**Fig 1:**
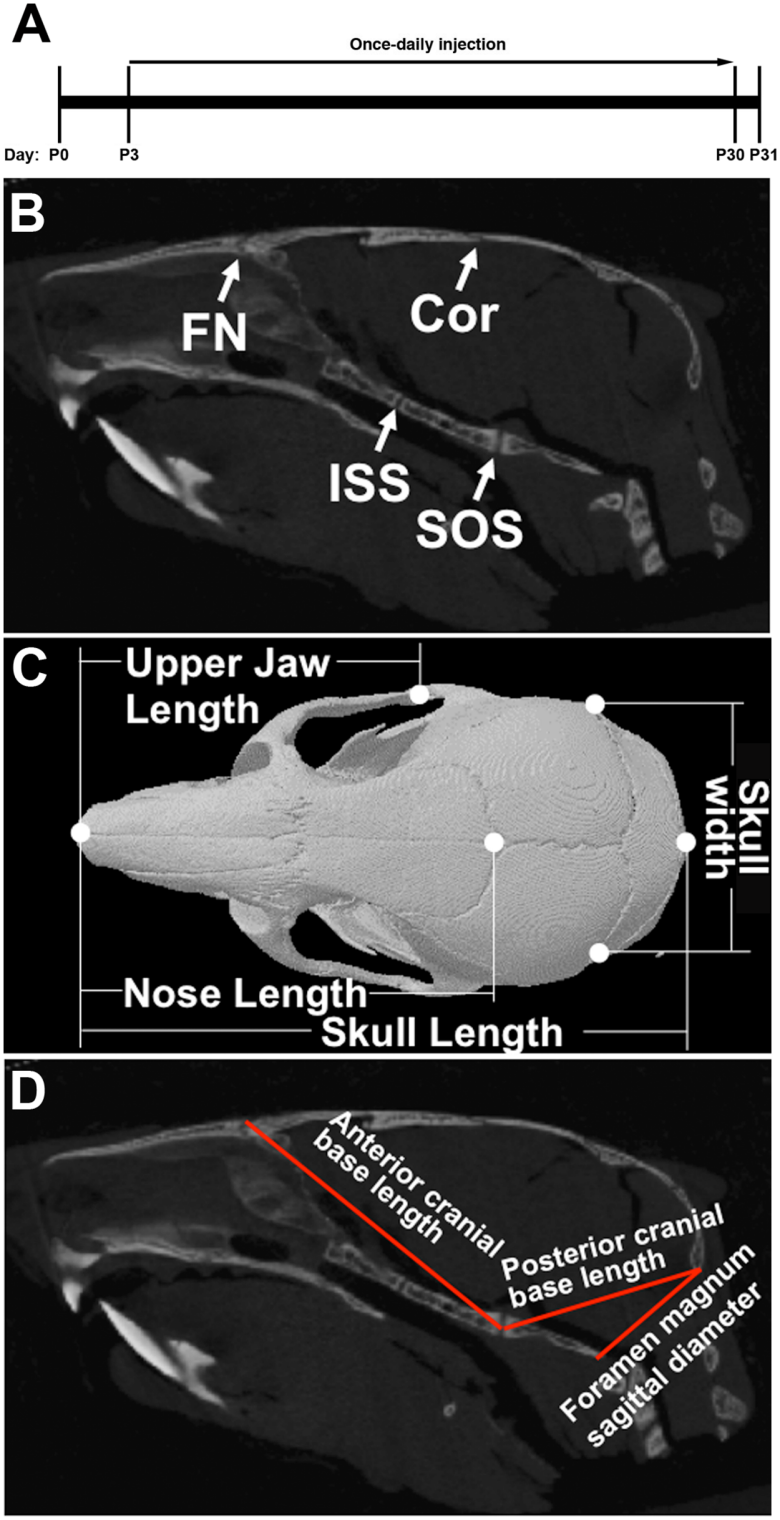
Locations of sutures and linear dimensions analyzed in the skull. (A) BMN 111 treatment timeline. (B) 2D sagittal slice of μCT data showing the coronal (Cor) and frontonasal (FN) sutures and the intersphenoidal (ISS) and spheno-occipital (SOS) synchondroses. (C) 3D reconstruction of μCT data showing a dorsal view of the skull with skull, nose, and upper jaw lengths and skull width indicated. (D) 2D sagittal slice of μCT data showing the anterior and posterior cranial base lengths and the foramen magnum sagittal diameter.

For the *in vivo* evaluation of BMN 111, male and female WT and mutant mice were divided into 6 treatment groups, each with a minimum of 10 mice, with approximately equal numbers of males and females. Brachycephaly and extensive coronal suture fusion does not appear in this mouse model until two weeks of age [25]. To test the effect of BMN111 postnatally we elected to initiate treatment from postnatal day (P) 3 and throughout the postnatal period until P30 to test whether BMN 111 can reduce or prevent skull dysmorphology and sutural fusion. The study design is outlined in Table 1, Fig 1A, and described below.

**Table 1:** Study design for evaluation of BMN 111 in *Fgfr2*^*+/+*^ and *Fgfr2c*^*C342Y/+*^ mice.

Group	Treatment	Treatment duration	Termination
1, 2	None	None	P3
3, 4	Vehicle	28 days (P3 to P30)	P31
5, 6	BMN 111	28 days (P3 to P30)	P31

Groups 1 and 2 consisted of untreated *Fgfr2*^*+/+*^ and *Fgfr2c*^*C342Y/+*^ mice, respectively, sacrificed at P3 to provide a baseline control for the frequency of suture and synchondrosis fusion. Groups 3 and 4 consisted of *Fgfr2*^*+/+*^ and *Fgfr2c*^*C342Y/+*^ mice, respectively, treated once daily with vehicle for 28 days, from P3 to P30, and sacrificed at P31. Groups 5 and 6 consisted of *Fgfr2*^*+/+*^ and *Fgfr2c*^*C342Y/+*^ mice, respectively, treated once daily with BMN 111 in vehicle for 28 days, from P3 to P30, and sacrificed at P31.

Vehicle consisted of 5 mM citrate, pH5.5, 5.8% (w/v) trehalose dehydrate, 1.5% (w/v) mannitol, 0.727 mg/ml methionine, and 0.005% (w/v) polysorbate 80. BMN 111 was prepared at a concentration of 80 μg/ml in vehicle and administered at 10 μL/g body weight, for a final dosage of 800 μg/kg/day [18], by subcutaneous injection at the midscapular region. Care was taken to ensure that injected solution was not expelled from the injection site. Doses were given at approximately the same time each day, +/- 2 hours of the first day’s dose time, with volume based on the body weight determined each day before injection.

The sex and *Fgfr2* genotype for each pup was determined from tail biopsy samples collected at P2. DNA was extracted and genotyping performed by polymerase chain reaction (PCR) amplification to confirm the absence or presence of the *Fgfr2c*^*C342Y/+*^ heterozygous mutation [24]. At the time of tail biopsy pups were tattooed on the paws with a unique identifying code. Pups were assigned manually to treatment groups, with males and females of each genotype in a given litter distributed as equally as possible to each treatment group. Groups 1 and 2 were obtained from 3 litters. Groups 3–6 were obtained from 5 litters. Upon sacrifice by isoflurane inhalation, P3 mice were fixed in 10% neutral-buffered formalin for 48 hours followed by transfer to PBS/0.1% sodium azide for long-term storage; P31 mice were treated similarly but were skinned and eviscerated before fixation.

### Micro-computed tomography analysis

Skulls from treatment groups 1–6 and tibia from treatment groups 3–6 were scanned using the SkyScan 1176 Device (Bruker, Belgium) with a resolution of 18 μm for skull and 35 μm for tibia. The X-ray source settings were set to 50kV, 500 μA and a 0.5 mm Al filter was used. Images were reconstructed using NRecon software (Bruker, Belgium).

To measure linear skull dimensions and determine suture patency, images were processed by DataViewer (Bruker, Belgium). The optimal bone position was determined by viewing the 3D reconstruction and two-dimension individual slices in the orthogonal views (including coronal, transaxial, and sagittal planes). For measuring linear skull dimensions multiple landmarks were placed and the lengths of skull, nasal, upper jaw, skull width, foramen magnum sagittal diameter, anterior cranial base length, and posterior cranial base length were obtained using DataViewer (Fig 1B–1D). To determine suture patency, the individual sutures were viewed throughout the length of the entire suture in three planes (coronal, transaxial, and sagittal). The assessment of patency of the coronal and frontonasal sutures and the spheno-occipital and intersphenoidal synchondroses was adapted from a previously described method [26]. Briefly, all sutures were scored qualitatively as “patent” when the suture was completely patent; “early fusion” when less than 25% of the length of the suture was fused; “partial” when more than 25% but less than 75% of the length of the suture was fused; or “fused” when more than 75% of the length of the suture was fused. In mice used in this study, no sutures or synchondroses in the “early fusion” category were observed.

To validate successful delivery and treatment effects of BMN 111, tibia length of animals from treatment groups 3–6 were measured as a pharmacodynamic marker, based on previous reports where pharmacologic effects of BMN 111 were observed at the dose concentration used for the present study [18, 19].

All measurements of suture patency, skull dimensions, and tibia are provided in S1 Table. For data analysis, both sexes of mice were pooled for each group as no differences had previously been reported [26]. GraphPad Prism 6 was used to create graphs and determine statistical significance via one-way ANOVA with post hoc Tukey analysis for length parameters and chi-square test for the percentage of suture patency. Results were plotted in GraphPad Prism 7 as box-and-whisker plots. Whiskers indicate maximum and minimum values.

## Results

**Fig 2:**
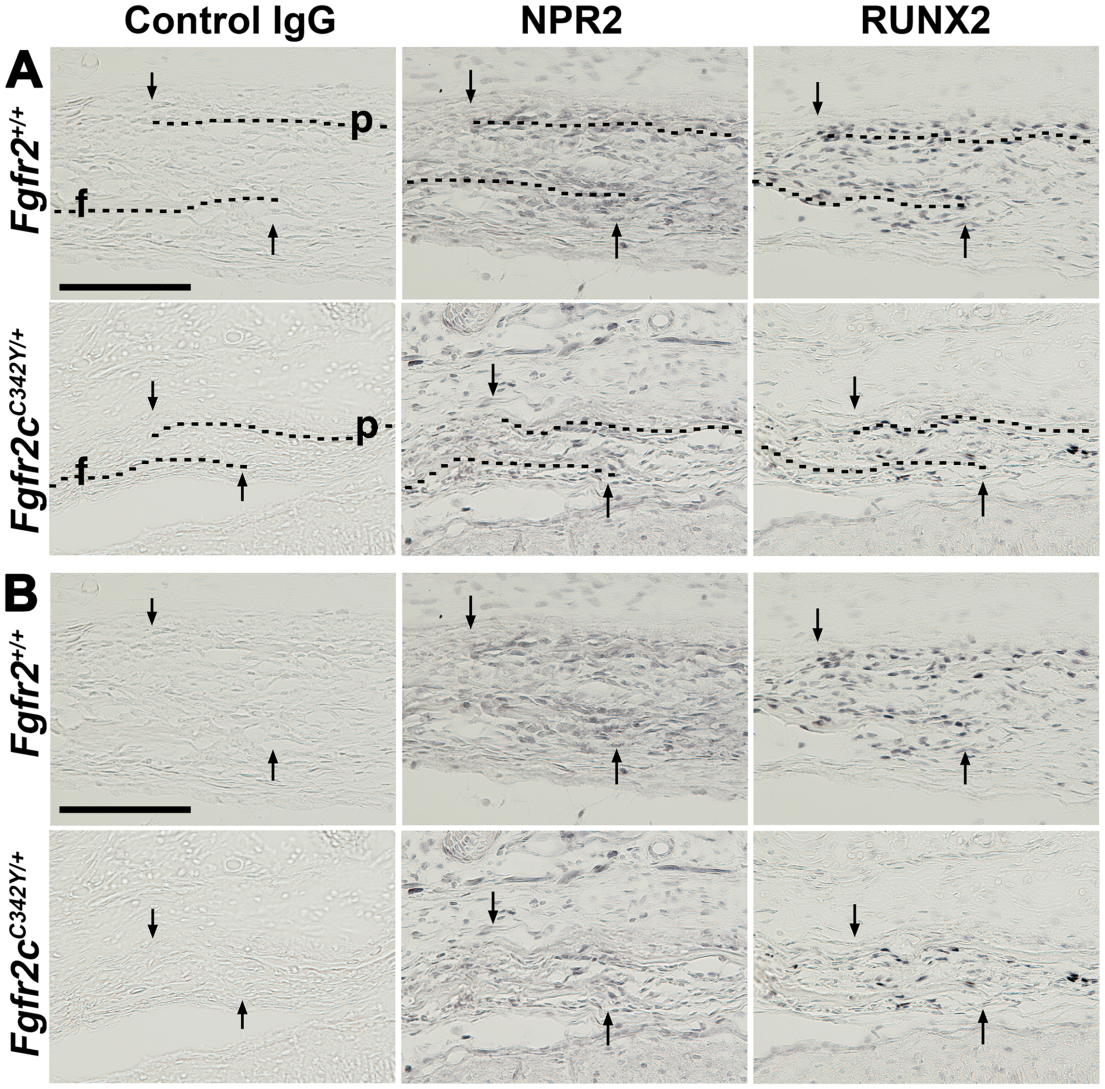
Immunohistochemical detection of NPR2 expression in the coronal suture at P0. Near-adjacent frozen sections were incubated with control IgG serum or antibodies to NPR2 or RUNX2. (A) For orientation, dashed lines overlay the osteoid of frontal (f) and parietal bones (p) and the arrows indicate the osteogenic fronts of these bones. Sagittal sections, oriented with anterior to the left. (B) Same sections as in (A) are shown without dashed lines over the osteoid for clearer visualization. In the coronal suture NPR2 expression is present in osteoblasts along the osteoid of the frontal and parietal bones. RUNX2 is expressed predominantly in the osteoblasts. No signal was seen with control IgG serum. Scale bars are 200 μm.

**Fig 3:**
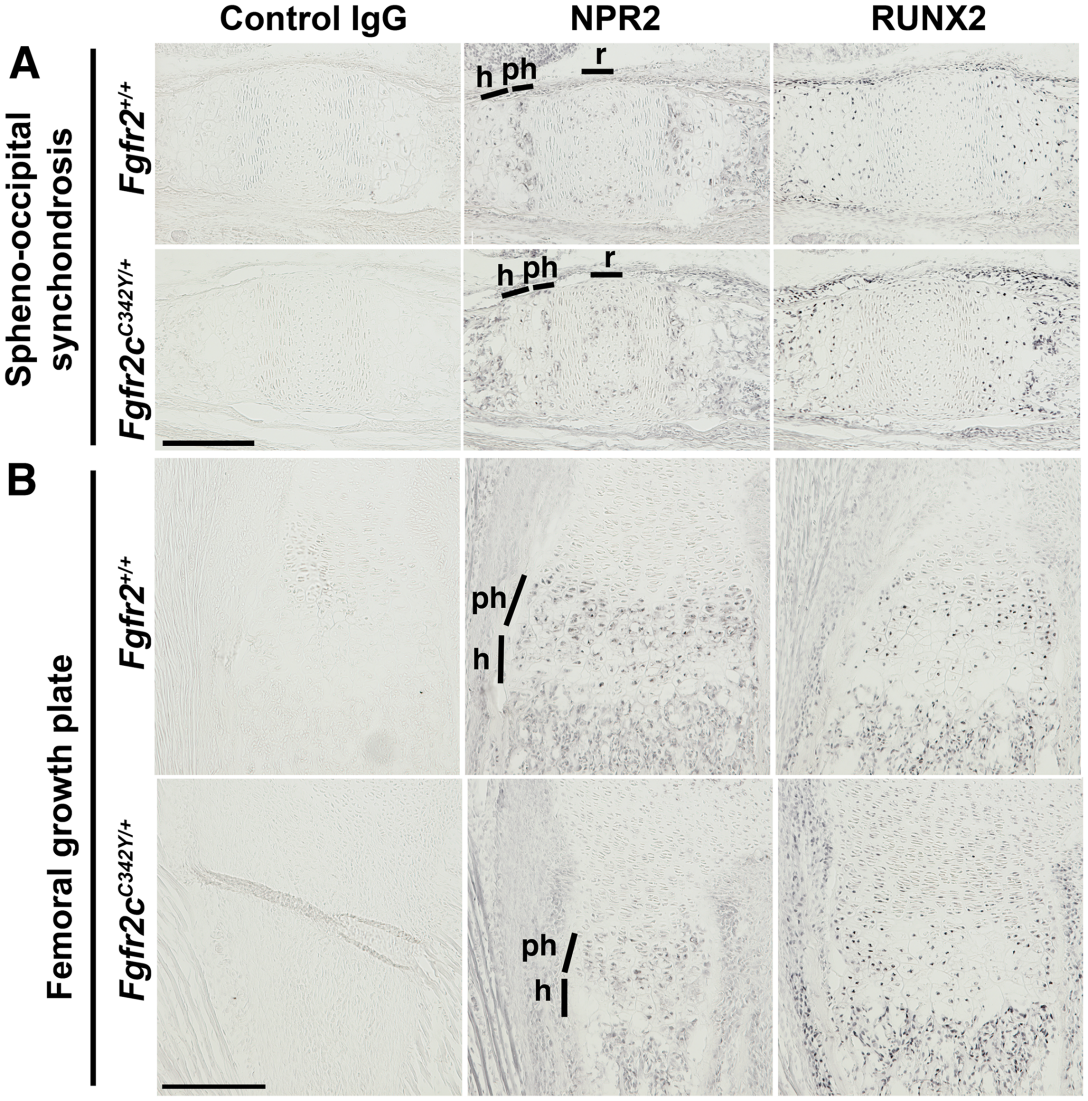
Immunohistochemical detection of NPR2 expression in growth plates at P0. Near-adjacent frozen sections were incubated with control IgG serum or antibodies to NPR2 or RUNX2. (A) In the spheno-occipital synchondrosis, NPR2 expression was higher in the prehypertrophic (ph) than the hypertrophic (h) zone, with some expression in the resting (r) zone. RUNX2 was expressed less in the prehypertrophic than the hypertrophic zone. Sagittal sections, oriented with anterior to the left. (B) In the proximal femoral growth plate, NPR2 expression was present in the prehypertrophic and hypertrophic zones, similar to RUNX2 expression. Transverse section. No differences were seen between *Fgfr2*^*+/+*^ and *Fgfr2c*^*C342Y/+*^ mice. No signal was seen with control IgG serum. Scale bars are 200 μm.

BMN 111 has previously been shown to partially correct the long bone growth plate defect in 7- or 21-day old *Fgfr3* mouse models of achondroplasia [18, 19]. Cartilaginous growth plates of long bones and the cranial base express *Fgfr-1*, *-2*, *-3* and *Npr2* [6, 7, 18, 27–29]. Osteoblasts within the calvarial sutures express *Fgfr-1*, *-2*, *-3* [30], but *Npr2* expression within sutures has not been characterized. We first determined whether NPR2 protein is expressed in the coronal suture by immunohistochemistry prior to testing whether BMN 111 could act through NPR2 to ameliorate cranial dysmorphology in Crouzon mice. NPR2 was expressed in the newborn period as early as P0 in osteoblasts of the coronal suture (Fig 2). NPR2 was also expressed in prehypertrophic cells of the spheno-occipital synchondrosis at P0 (Fig 3A), in accordance with a previous report of expression at P14 [7], and at similar levels of localization and staining intensity in both WT and mutant mice. We also verified that NPR2 was expressed in proliferating and prehypertrophic cells of the femoral growth plate (Fig 3B) in both WT and mutant mice, as previously reported for long bones of WT mice [6, 18, 19].

**Fig 4:**
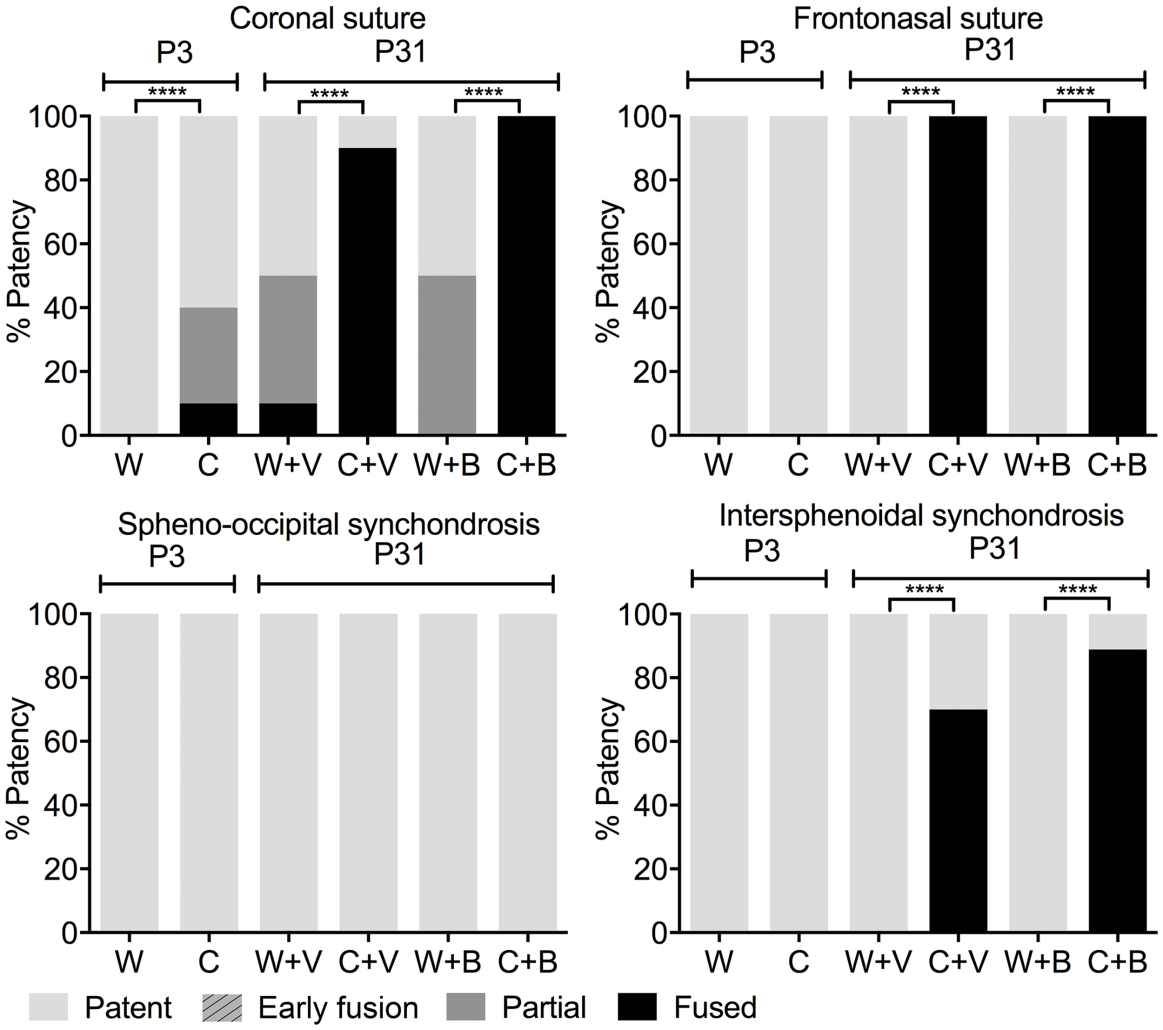
Patency of craniofacial sutures and effect of BMN 111. Graphs show patency of the coronal and frontonasal sutures and the spheno-occipital and intersphenoidal synchondroses at P3 (no treatment) and P31 (treatment of wild type [W] or Crouzon mutant mice [C] with either BMN 111 [B] or vehicle alone [V]). No sutures or synchondroses in the “early fusion” category were observed.

We assessed the ability of BMN 111 to alleviate the Crouzon craniofacial phenotype following subcutaneous injection for 28 days (Fig 1A). Skulls and tibia were fixed and scanned by micro-computed tomography (μCT). Untreated WT and mutant mice at P3 were similarly fixed and scanned to provide a baseline control for the experiment. We first assessed patency of the calvarial coronal and frontonasal sutures, and the cranial base spheno-occipital and intersphenoidal synchondroses (Fig 1B). At P3 all sutures and synchondroses were patent in both WT and mutant mice, except for the coronal suture of mutant mice, which was partially or completely fused in 40% of mice (Fig 4, S1 Table). At P31 in WT mice, the frontonasal suture, and spheno-occipital and intersphenoidal synchondroses remained patent, with no differences seen between mice treated with either BMN 111 or vehicle alone. The WT coronal suture was partially or completely fused in 50% of mice, with no clear difference between treatment with either BMN 111 or vehicle alone. In contrast, at P31 in mutant mice, the frontonasal suture was completely fused in all mice, the coronal suture was fused in 90% of mice, and the intersphenoidal synchondrosis was completely fused in 70% of mice. The spheno-occipital synchondrosis was not fused in mutant mice. No clear difference in the incidence of suture fusion was apparent between treatment with either BMN 111 or vehicle alone.

**Fig 5:**
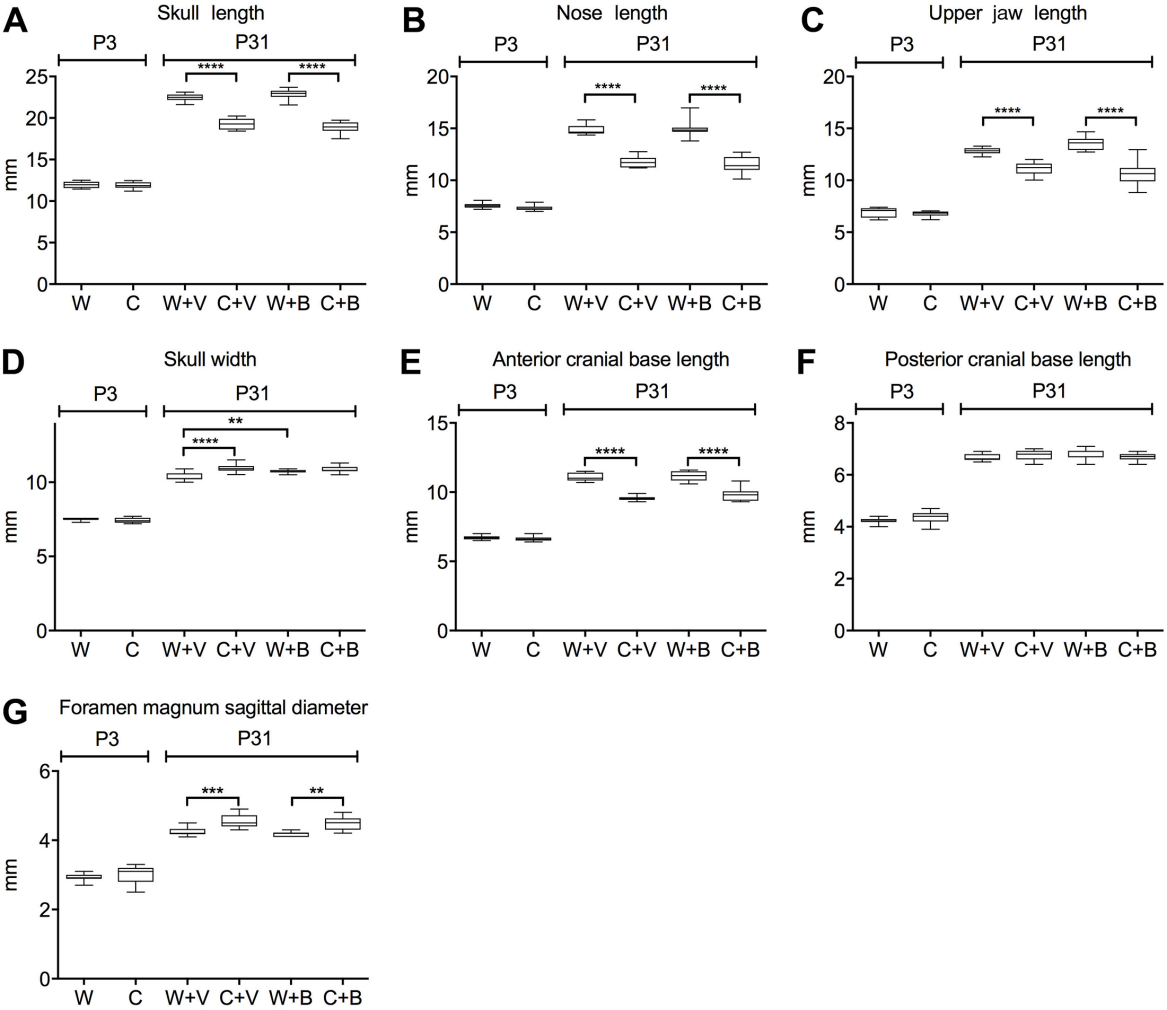
Linear dimensions of the calvaria and skull base and effect of BMN 111. Graphs show the indicated linear dimensions in millimeters (mm) at P3 (no treatment) and P31 (treatment of wild type [W] or Crouzon mutant mice [C] with either BMN 111 [B] or vehicle alone [V]). (A) Skull length. (B) Nose length. (C) Upper jaw length. (D) Skull width. (E) Anterior cranial base length. (F) Posterior cranial base length. (G) Foramen magnum sagittal diameter. **p<0.01, ***p<0.001, ****p<0.0001.

We next assessed changes in intramembranous and endochondral skull growth by measuring various linear dimensions between landmarks of the craniofacial bones (Fig 1C) or the cranial base (Fig 1D). At P3 no differences were seen between WT and mutant mice in the lengths of the skull, nose, or jaw; width of skull; length of the anterior or posterior cranial base; or sagittal diameter of the foramen magnum (Fig 5A–5G, S1 Table). By P31 mutant mice clearly differed from WT mice with mutant mice having significantly shorter skulls, noses, and upper jaw lengths (Fig 5A–5C), but a greater skull width (Fig 5D). Mutant mice had a significantly shorter anterior cranial base (Fig 5E), while the posterior cranial base length did not differ from the WT (Fig 5F). The foramen magnum sagittal diameter was significantly greater in mutant mice compared to WT (Fig 5G). Treatment with BMN 111 generally had no effect on these dimensions in either WT or mutant mice (Fig 5A–5G), except for a slight increase in skull width of WT mice (Fig 5D). We further analyzed the effect of BMN 111 treatment on these measurements by comparing the ratio of each linear length to skull length between vehicle- and BMN 111-treated mice (Table 2). For WT mice, no significant differences were seen. For Crouzon mice, a significant difference was seen for the anterior skull base, which increased with BMN 111 treatment. These results show that while overall there was no strong effect on skull size with BMN 111 treatment for either genotype, small effects on skull dimensions could be seen.

**Table 2:** Cranial and tibial linear measurements normalized by skull length.

Linear measurements normalized by skull length	W+V^a^	C+V^a^	W+B^a^	C+B^a^	P value^b^	
					W+V vs W+B	C+V vs C+B
Upper jaw length	0.571±0.004	0.579±0.012	0.593±0.007	0.565±0.016	0.056	0.914
Nose length	0.659±0.004	0.611±0.008	0.655±0.011	0.609±0.012	0.991	0.999
Skull width	0.461±0.004	0.567±0.007	0.468±0.003	0.577±0.007	0.585	0.829
Anterior cranial base length	0.492±0.002	0.495±0.004	0.488±0.004	0.520±0.006	0.795	0.021
Posterior cranial base length	0.297±0.002	0.351±0.004	0.298±0.001	0.355±0.004	0.972	0.808
Foramen magnum sagittal diameter	0.190±0.002	0.237±0.005	0.183±0.002	0.238±0.006	0.092	0.999
Tibia length	0.706±0.004	0.796±0.011	0.735±0.005	0.859±0.007	0.006	0.003

W, wild type; C, Crouzon; V, vehicle; B, BMN 111.

^a^Values are mean ± SEM;

^b^P values determined by one-way ANOVA with post hoc Tukey analysis.

**Fig 6:**
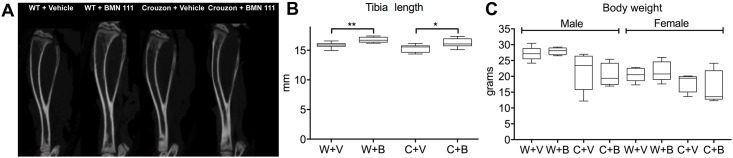
Tibia length and effect of BMN 111. (A) 2D slice of μCT data showing tibias at P31. (B) Graph shows the tibia length in millimeters (mm) at P31, males and females combined, after treatment of wild type [W] or Crouzon mutant mice [C] with either BMN 111 [B] or vehicle alone [V]. (C) Graph shows body weight at P31 for males and females, after treatment of wild type [W] or Crouzon mutant mice [C] with either BMN 111 [B] or vehicle alone [V]. *p<0.05, **p<0.01.

Previous studies showed that BMN 111 increased long bone growth in both WT mice and mutants from *Fgfr3*^*Y367C/+*^ thanatophoric dysplasia [18] and *Fgfr3*^*ACH/+*^ achondroplasia [19] mouse models. We therefore measured tibia length as a pharmacodynamic marker to verify the biological activity of BMN 111 in our experiments. Tibia length was significantly increased in both WT and mutant mice, by 5.8% and 5.6%, respectively, consistent with previous studies (Fig 6A and 6B, S1 Table). Tibia lengths were also normalized to skull lengths and the ratios were compared between vehicle- and BMN 111-treated mice for each genotype. Tibia length was significantly increased relative to skull length in both WT and mutant mice after treatment with BMN 111 (Table 2). Body weights of WT and mutant male and female mice were not significantly different after treatment with BMN 111 (Fig 6C). Therefore, tibial length increase was not due to a general increase in skeletal or body size.

## Discussion

In previous studies of thanatophoric dysplasia and achondroplasia mouse models carrying activating FGFR3 mutations, BMN 111 was shown to improve the endochondral development of the growth plate and subsequent growth of long bones [18, 19]. Cranial morphology also is affected in both thanatophoric dysplasia and achondroplasia mouse models. The thanatophoric dysplasia mice have a shorter, dome-shaped skull, shorter snout, and anterior crossbite [18, 31, 32]. In this animal model, with BMN 111 treatment there was morphological improvement, with flattening of the skull, elongation of the snout, and reduced crossbite [18]. However, the areas of change in either the intramembranous calvarial bones or endochondral base of the skull were not assessed quantitatively [18]. The achondroplasia mice have a shorter, dome-shaped skull and a shorter mandible [13]. Skull development was not evaluated in the BMN 111-treated achondroplasia mouse model, but as expression of the mutant FGFR3 is targeted only to cartilage using a collagen 2 promoter sequence, effects on intramembranous skull bones that do not develop from cartilage are secondary. Thus, this mouse model is not suitable to address the effect of BMN 111 on mutant intramembranous bone formation [19].

In this study we sought to determine the ability of BMN 111 to ameliorate *Fgfr2*-related dysplasias affecting both intramembranous and endochondral cranial bone formation. Crouzon syndrome mouse model mutants are relatively normal in appearance at birth, but develop noticeable brachycephaly by two weeks of age, and by four weeks of age the coronal sutures are almost completely fused [24, 25]. The shortened cranial base phenotype is common between Crouzon syndrome patients and this mouse model. In contrast, the sagittal diameter of the foramen magnum of Crouzon syndrome patients is smaller than normal, with fusion of the associated synchondroses early in childhood [33], while in the mouse model this diameter is larger than normal by P31, and the associated synchondroses are still patent around this age [24]. The mouse model is therefore not appropriate for assessing potential improvements of the human foramen magnum phenotype.

We treated both WT and mutant mice with once-daily injections of either BMN 111 or vehicle alone between P3 and P31. At P3 there is some degree of fusion in mutant coronal sutures, but the frontonasal suture and spheno-occipital and intersphenoidal synchondroses are patent, and no difference in the lengths of the skull, nose, or jaw; width of skull; length of the anterior or posterior cranial base; or sagittal diameter of the foramen magnum are seen between WT and mutant mice (Fig 5). Treatment with BMN 111 from P3 therefore is appropriately timed to reduce or prevent the incidence of mutant phenotypes. No significant effect of BMN 111 in mutant mice was seen on the incidence of coronal or frontonasal suture fusion; length of the skull, nose, and jaw; or width of the skull (Fig 5). BMN 111 showed clear pharmacological activity on endochondral bone growth, as seen by the significant increase in tibia length in both WT and mutant mice (Fig 6), but the length of the anterior and posterior cranial bases and the sagittal diameter of the foramen magnum were not affected (Fig 5). However, in Crouzon mice the ratio of the anterior cranial base length to skull length did increase slightly but significantly, suggesting a subtle effect on endochondral growth in the cranial base (Table 2). WT skull width also increased slightly but significantly with BMN 111 treatment (Fig 5). Normally, skull width increases most significantly during P7-P14 [34], which falls within our treatment period. BMN 111 therefore may affect FGFR signaling that regulates intramembranous or endochondral growth mechanisms affecting skull width during this time. Crouzon mutant mice have a wider skull than WT mice at P31, but this width was not increased further with BMN 111 treatment.

The dosage of BMN 111 used to treat the Crouzon mice in the present study (800 μg/kg/day) was the same as the maximum effective dosage used for the thanatophoric dysplasia *Fgfr3*^*Y367C/+*^ mouse model, in which mice were treated for up to 20 days from P7, a comparable time period to that used in our study [18]. The *Fgfr3*^*Y367C*^ mutation is one of the more strongly activating mutations of *Fgfr3* causing chondrodysplasia, generating a high basal level of the downstream signaling effector, phospho-ERK [35, 36]. Similarly, the *Fgfr2c*^*C342Y*^ mutation is one of the more strongly activating mutations of *Fgfr2* causing craniosynostosis, being ligand-independent and generating a higher basal level of phospho-ERK compared to, for example, Apert syndrome mutations [35]. The BMN 111 dosage used in this study may not have been sufficient to inhibit mutant receptor activity, but may be efficacious against more weakly activating *Fgfr2* mutations than the *Fgfr2c*^*C342Y*^ mutation.

By inhibiting RAF1 kinase, BMN 111 treatment should affect signaling from all four FGFRs. However, the FGFR signaling pathway is highly complex, with multiple levels of regulation, feedback inhibition, and cross-talk with other signaling pathways [3]. Signaling from individual FGFRs therefore may vary in sensitivity to the effects of BMN 111. In addition, the specific activating FGFR mutations may affect the downstream signaling pathways in ways that impact sensitivity to inhibition by BMN 111. These issues require further exploration.

In summary, BMN 111 can affect skeletal growth by the inhibition of FGFR signaling. Its efficacy in ameliorating skeletal dysplasias varies, and may depend on such factors as the target tissue, the specific FGFR involved, and the receptor mutation.

## Supporting information

S1 Table Measurements of suture patency, skull dimensions, and tibia. (XLSX)
